# A home-made pipette droplet microfluidics rapid prototyping and training kit for digital PCR, microorganism/cell encapsulation and controlled microgel synthesis

**DOI:** 10.1038/s41598-023-27470-1

**Published:** 2023-01-05

**Authors:** Liao Chen, Chenguang Zhang, Vivek Yadav, Angela Wong, Satyajyoti Senapati, Hsueh-Chia Chang

**Affiliations:** Department of Chemical and Biomolecular Engineering, University of Notre Dame, Notre Dame, IN 46556 USA

**Keywords:** Analytical biochemistry, Lab-on-a-chip, Techniques and instrumentation, Biomedical engineering, Chemical engineering, Chemical education

## Abstract

Droplet microfluidics offers a platform from which new digital molecular assay, disease screening, wound healing and material synthesis technologies have been proposed. However, the current commercial droplet generation, assembly and imaging technologies are too expensive and rigid to permit rapid and broad-range tuning of droplet features/cargoes. This rapid prototyping bottleneck has limited further expansion of its application. Herein, an inexpensive home-made pipette droplet microfluidics kit is introduced. This kit includes elliptical pipette tips that can be fabricated with a simple DIY (Do-It-Yourself) tool, a unique tape-based or 3D printed shallow-center imaging chip that allows rapid monolayer droplet assembly/immobilization and imaging with a smart-phone camera or miniature microscope. The droplets are generated by manual or automatic pipetting without expensive and lab-bound microfluidic pumps. The droplet size and fluid viscosity/surface tension can be varied significantly because of our particular droplet generation, assembly and imaging designs. The versatility of this rapid prototyping kit is demonstrated with three representative applications that can benefit from a droplet microfluidic platform: (1) Droplets as microreactors for PCR reaction with reverse transcription to detect and quantify target RNAs. (2) Droplets as microcompartments for spirulina culturing and the optical color/turbidity changes in droplets with spirulina confirm successful photosynthetic culturing. (3) Droplets as templates/molds for controlled synthesis of gold-capped polyacrylamide/gold composite Janus microgels. The easily fabricated and user-friendly portable kit is hence ideally suited for design, training and educational labs.

## Introduction

Droplet microfluidics has emerged as a powerful tool for a variety of multidisciplinary applications^1,2^. Exciting commercialized and proposed biotech technologies based on droplet microfluidics include single-cell assay^3,4^, droplet digital PCR^5,6^, rapid cancer screening^7,8^, drug or immunotherapy screening^9,10^, antibiotic susceptibility screening^11^, implantable islet allografts^12,13^ etc. Generally, for those and many other applications, droplets are typically used as microreactors, microcompartments or templates/molds and large numbers of them guarantee the unique advantages of droplet microfluidics^14–17^. A good example is the droplet digital polymerase chain reaction (ddPCR) which uses droplets as microreactors^6,18^. Simply by dividing the PCR reaction mix into many uniform droplets, the target nucleic acids are distributed into the droplets according to the Poisson distribution and can be subsequently quantified to single-copy resolution after PCR amplification^19^. The improved performance is enhanced by a large number of droplets (10^4^–10^6^) which mitigates the influences of inhibitors or non-targets and permits absolute quantification without tedious construction of standard curves^20,21^. Another example is cell/microorganism encapsulation in droplets or droplet-derived microgels which could provide numerous microcompartments with the proper biophysical/biochemical microenvironments for high throughput study of isolated single cells and microorganisms to expedite large-scale screening and optimization even with sequential assay analysis permitted by permeable microgels^13,22–28^. In addition to microreactors and microcompartments applications, droplets as micro templates/molds have been suggested for the synthesis of soft and hard micro-/nano-materials^29–31^. The controlled miniature size, large surface area per unit volume, confinement, and large numbers of the droplets enable facile synthesis of materials with desired structures and functionalities because of rapid segregation and concentration effects introduced by high hydrophobicity gradient, strong surfactant/surface interaction and crowding effects^32,33^. Droplet microfluidics has clearly inspired several promising technologies in numerous disciplines.

**Figure 1:**
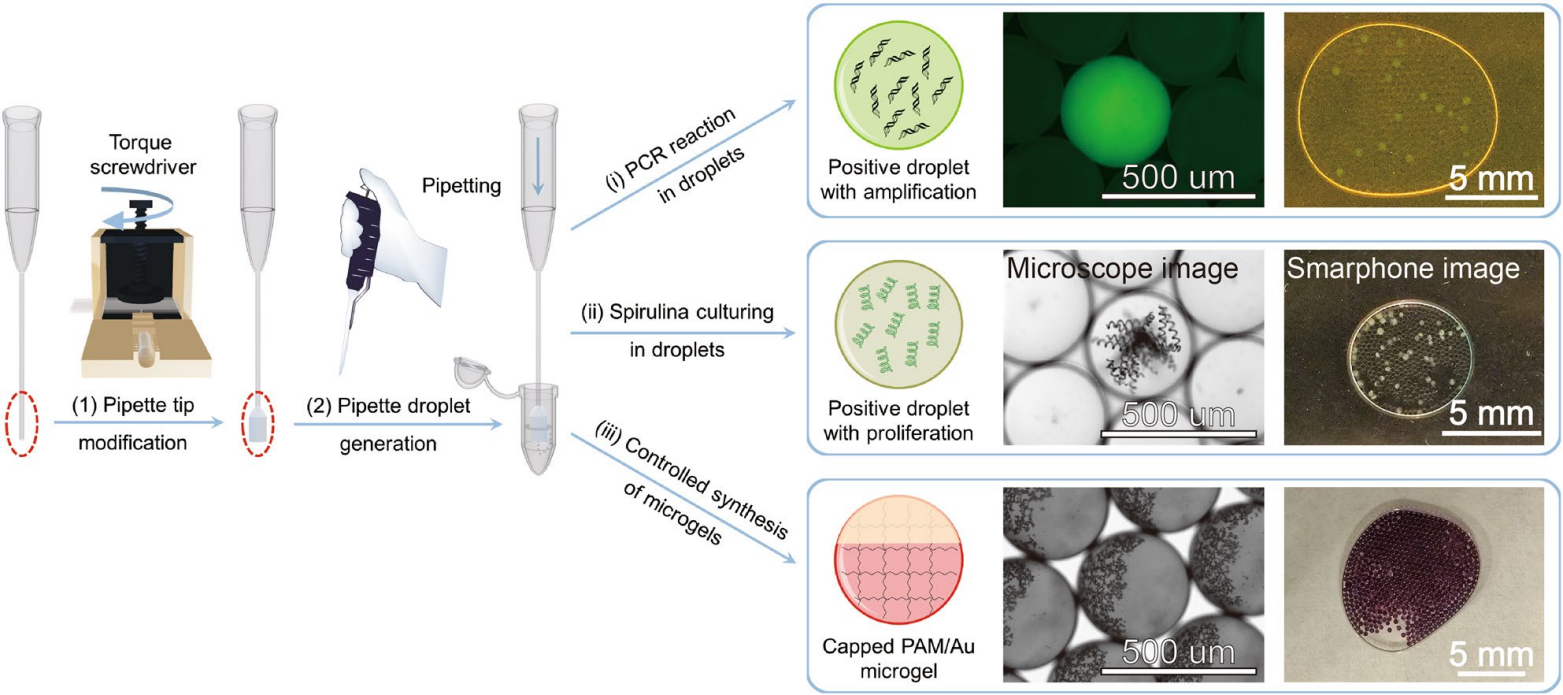
Pipette droplet microfluidics kit: (1) Schematic illustration of controllable pipette tip modification by a torque screwdriver tool results in deformed commercial pipette tips with flattened head parts. The modification will change the original circular orifice of the pipette tip to an elliptical one and the aspect ratio of the elliptical orifice is determined by the torque force from the screwdriver. (2) Droplet generation with the modified tips via a universal micro pipettor. Uniform droplets will pinch-off automatically when dispensing aqueous solution manually through an appropriately deformed pipette tip orifice into fluorinated oil. (i, ii, iii) Examples of typical droplet microfluidics applications about (i) droplet digital PCR with droplets as microreactors, (ii) culturing of spirulina with droplets as microcompartments, and (iii) controlled synthesis of microgels with droplets as the templates/molds.

However, current droplet generation systems^1,14,34^, which often require sophisticated chips/devices and/or precision pressure sources (that cost over five thousand dollars for commercial droplet generation kits from PreciGenome, Elveflow, Fluigent etc.), are not sufficiently simple, cheap and accessible to allow easy prototyping and training, thus limiting the broad implementation of droplet microfluidic technologies in interdisciplinary fields and further development of new technologies into commercializable products. For example, the widely used flow-focusing droplet generation technology needs fine tuning with accurate control of two-phase flows in microfluidics chips^35^ and the relatively flow-rate-insensitive step emulsion droplet generation usually needs carefully designed chips/devices with terraces^36^. Besides uniform droplet generation, existing confinement-based droplet assembly and microscope-based imaging methods should also be simplified to be more accessible and customizable^6,37,38^. Therefore, a simple and rapid prototyping tool kit that includes a tunable microfluidic droplet generation technology, plus robust droplet assembly and imaging modules that are insensitive to droplet size and viscoelastic properties, would enable further expansion of its applications in interdisciplinary fields-much like what 3D printing has done for biochips and other branches of microfluidics^39^. To this end, based on our previously developed pipette droplet technology^40^, a standardized comprehensive pipette droplet microfluidics rapid prototyping and training kit is proposed in this work to allow not only easily tunable droplet features but also robust and inexpensive components that can assemble, immobilize and image droplets of different size and with different rheological and surface properties. (Fig. 1).

The versatility of the robust kit is due to three key technologies that can be easily and rapidly fabricated to allow maximum tunability. Uniform microdroplets are conveniently generated by pipetting liquids out from pipette tips with elliptical orifices for enhanced droplet pinch-off. The elliptical pipette tips are prepared by deforming commercial round-orifice pipette tips with a DIY torque screwdriver tool and the size of the droplets can be adjusted by simply changing the orifice aspect ratio of the deformed pipette tip. The droplets are packed and immobilized against the shallow center of an easily made double-sided tape chip by a robust droplet assembly mechanism based on capillary pressure-driven wetting flow. The droplet suspension approaches a stable axisymmetric geometry with a circular meniscus and confines the droplets to a concentric circular monolayer at the center. Droplet imaging is simplified because of the large and immobilized microdroplets (300–500 microns in diameter) generated by this kit, such that the droplets can be imaged by smartphone cameras or observed visually. In terms of cost, the DIY screwdriver-based tip modification tool is less than a hundred dollars, the gel-loading tip is less than a dime per tip, a micro pipettor is two or three hundred dollars, the imaging chip made of a glass slide and several pieces of double-sided tape is less than a quarter, and imaging can be done by a regular hundred-dollar smartphone (an one-thousand-dollar transilluminator or handheld fluorescent microscope is needed for fluorescence imaging). The entire kit for droplet generation, handling, and imaging (including fluorescence imaging) can be realized with two thousand dollars, which is very cost-effective compared with the commercial kits costing over five thousand dollars for the droplet generation part alone. Moreover, micro pipettor, pipette tips, glass slides, double-sided Kapton polyimide tapes are common lab consumables and torque screwdrivers, smartphone cameras are common tools, which means the pipette droplet microfluidics kit proposed here is extremely accessible. The versatility of the kit is demonstrated with three prototyping applications of droplet microfluidics: (i) droplets as microreactors for droplet digital PCR to detect and quantify target RNAs; (ii) droplets as microcompartments for photosynthetic culturing of spirulina; (iii) droplets as templates/molds for controlled synthesis of gold-capped polyacrylamide/gold composite microgels. These typical examples demonstrate the versatility and user-friendliness of the pipette droplet microfluidics kit for rapid prototyping and validating droplet microfluidics applications.

## Results and discussion

### Tunable droplet generation via elliptical pipette tips prepared by home-made tool

**Figure 2:**
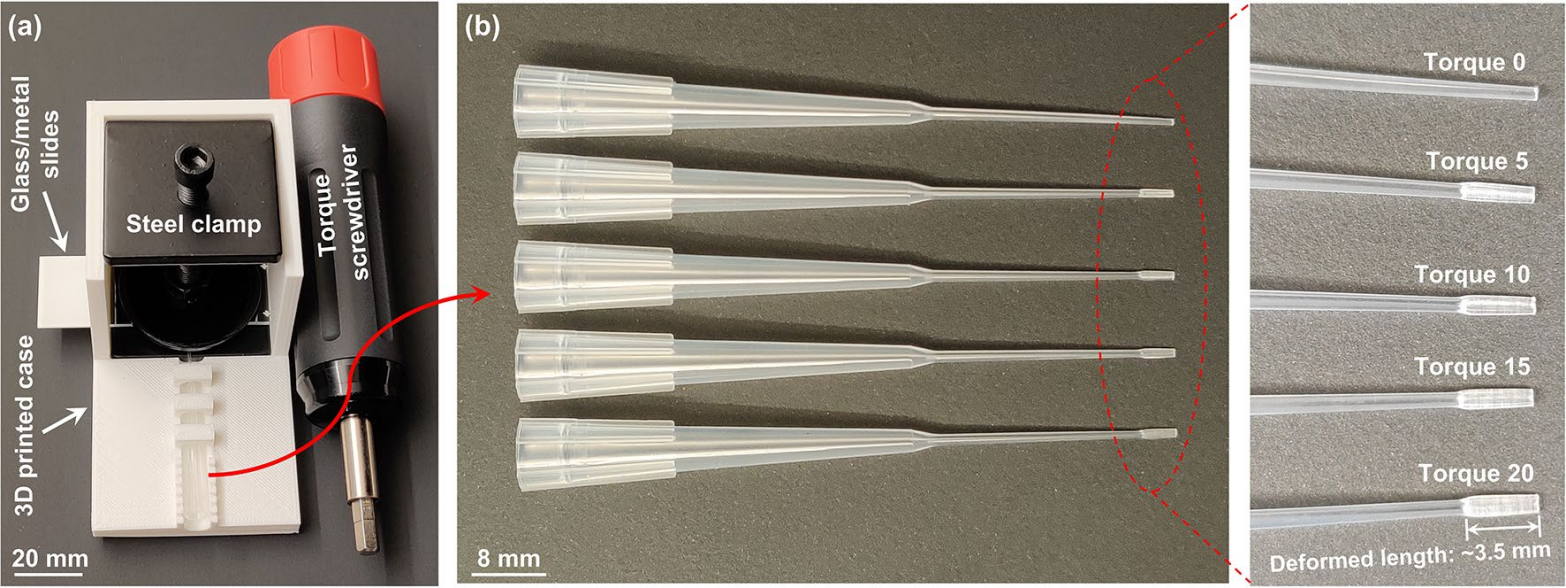
Standardized pipette tip modification to prepare head-flattened tips. (**a**) The simple tip deformation tool is easily assembled with a 3D printed case, two glass/metal slides, a steel clamp and a torque screwdriver. When using, a pipette tip is inserted between or under the two glass/metal slides. The glass/metal slides cannot rotate freely once assembled with the 3D-printed case and will transfer the torque force to pressure to deform the head part of the commercial pipette tip. (**b**) By setting the torque on the torque screwdriver from zero to twenty, modified pipette tips (deformed length: ~ 3.5 mm) with various degrees of deformation of the head parts are prepared.

**Figure 3:**
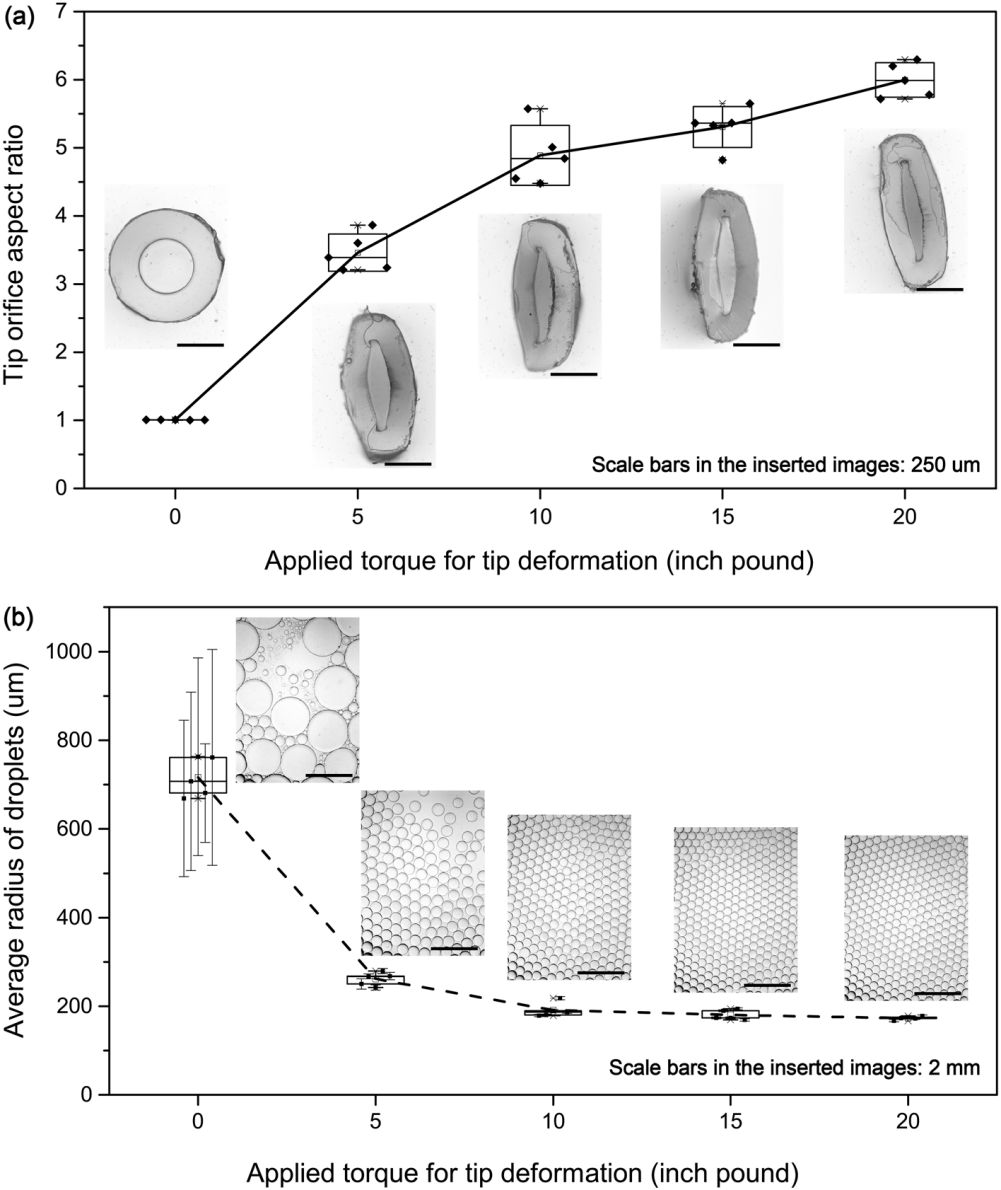
Controlled pipette tip modification by changing torque forces with constant deformed length (~ 3.5 mm) and corresponding pipette droplet generation: (**a**) cross-section aspect ratios of pipette tips versus applied torque for tip deformation, tip deformed lengths are ~ 3.5 mm, inserted images are typical deformed tip orifice cross-sections; (**b**) average radii of water droplets generated with torque deformed pipette tips, each point represents average radius and standard deviation (error bar) of droplets generated by the corresponding deformed tip and the inserted images are droplets generated by the tips with orifice cross-section images inserted in (**a**). Based on the curves, specific tips and droplets can be prepared by applying the corresponding torque forces.

In order to standardize tip modification, a series of torque forces (directly adjusted and set on a torque screwdriver) have been used to produce certain pressures to deform pipette tips with different elliptical orifices (Fig. 2, “Methods”: “Elliptical pipette tip preparation” section for more details about how to build and use the customized tool for tip fabrication). The relationship between aspect ratios of the tip orifices and the applied torque forces are shown in Fig. 3a. A calibration curve like this could provide guidance for making elliptical pipette tips with desired orifice aspect ratios for a specific type of tips prepared under same conditions. The home-made tool provides a channel to locate the tips and the interval barriers on the channel wall help determine the tip head length being deformed so that the elliptical tip fabrication conditions are more reproducible. (Fig. 2a) Moreover, the adjustment of deformed length (the length of the tip head part being deformed as indicated in Fig. 2b right) also allows another way to tune the degree of tip deformation (Fig. S1).

Deformed tips with different deformed lengths and/or different torque forces are characterized by checking the tip orifices (Figs. 2b, 3a, S1a,b, S2a,b). Under the same torque force, the shorter the tip head is there to share the pressure, the more deformed the tip head will be (Fig. S1b). Likewise, under the same deformed length, the higher the applied torque force, the more deformed the tip head will be (Fig. 3a, S2b). In addition, tips of different mechanical properties (due to different tip materials, orifice dimensions and wall thickness etc.) will behave and deform differently under pressure induced by the same torque force (Fig. S3a). As a result of this, for a new type of tips, a corresponding new calibration curve should be obtained via a standard protocol.

**Figure 4:**
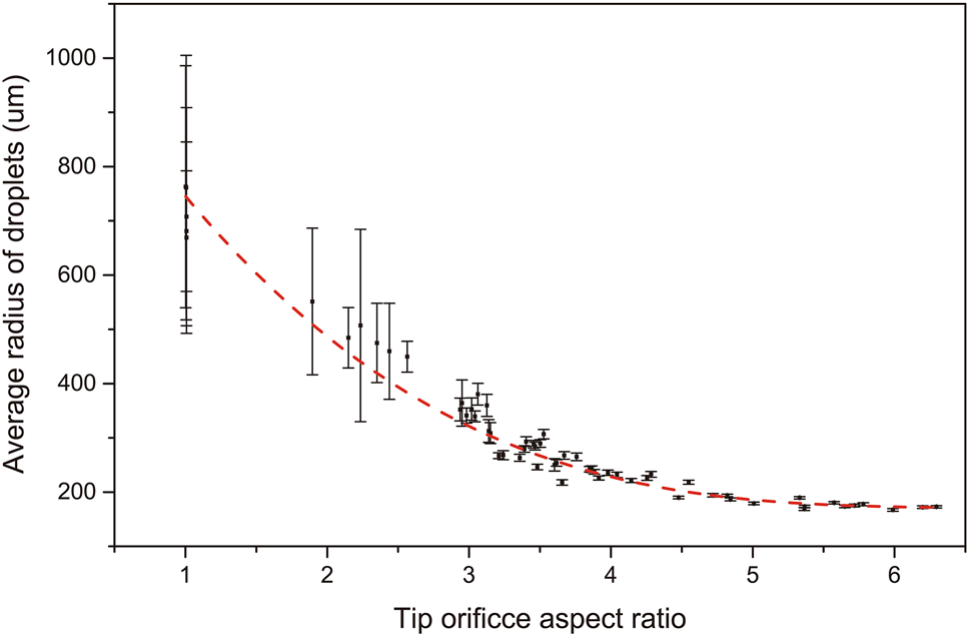
Water droplets generated by a series of deformed pipette tips (default 200 µL tips used in this work) with various tip orifice aspect ratios. Generally, for a certain type of tips, the higher the tip orifice aspect ratio, the more uniform the generated droplets.

The deformed pipette tips are equipped with a regular 20 µL pipettor for droplet generation. Technically, uniform droplets are generated in the dripping mode at very low flow rates^14^. But with deformed elliptical pipette tips, automatic pinch-off of droplets occurs even at high flow rate due to drainage of water from the pinching neck by the elevated neck water pressure. This elevated pressure is due to the amplified azimuthal curvature at the narrow end of the elliptical cross section. The enhanced neck capillary pressure results in more robust and faster generation of smaller uniform droplets similar to step-emulsion droplet generation from microfluidic chips with high-aspect ratio channels^41^ and the droplet generation is insensitive to moderate flow rate changes which facilitates manual pipetting to generate uniform droplets. (Video S1) As demonstrated in the figures (Figs. 3b, 4, S1c, S2c, S3b, S4), the higher the aspect ratio of the pipette tip orifice, the smaller and more uniform the generated droplets are. From aspect ratio of ~ 3.25, the coefficient of variation (CV) of generated droplets becomes less than 5% and from aspect ratio of ~ 4, the CV of generated droplets becomes less than 2%. The droplet uniformity is comparable to the commercial droplet generation systems, such as Elveflow droplet generation pack which claims a droplet CV of < 3%^42^. The large non-uniformities of droplets generated with low orifice aspect ratio tips are because of two reasons. First, the corresponding as-generated droplets are larger, and the larger droplets are easy to break up into smaller satellite droplets during droplet generation and handling. Second, the droplet generation with less elliptical tips requires lower flow rates and is more sensitive to disturbances during pipetting. The droplet range in Fig. 4 can be further expanded by using pipette tips of different orifice sizes (Fig. S5).

### Tape-based and 3D printed chips with designed shallow centers for monolayer droplets immobilization, assembly and imaging

Once the uniform droplets are generated, another prototyping challenge is the assembly and imaging of the droplets. The lighter water droplets tend to float to the top of the dispersed oil phase and would not assemble spontaneously to form monolayers for observation. Moreover, the wetting oil often carries the droplets away from the imaging position. These issues are typically resolved with a complicated enclosed chip design with pillars and other barriers to confine and pack droplets into monolayers^6,37,38^. Pinning the droplet in place against such barriers then requires a sustained back pressure and loading becomes a major microfluidic undertaking. Also, contact with the solid barriers often induces droplet coalescence and hence requires precise loading flow that must be tuned for droplets of different size and viscoelasticity.

**Figure 5:**
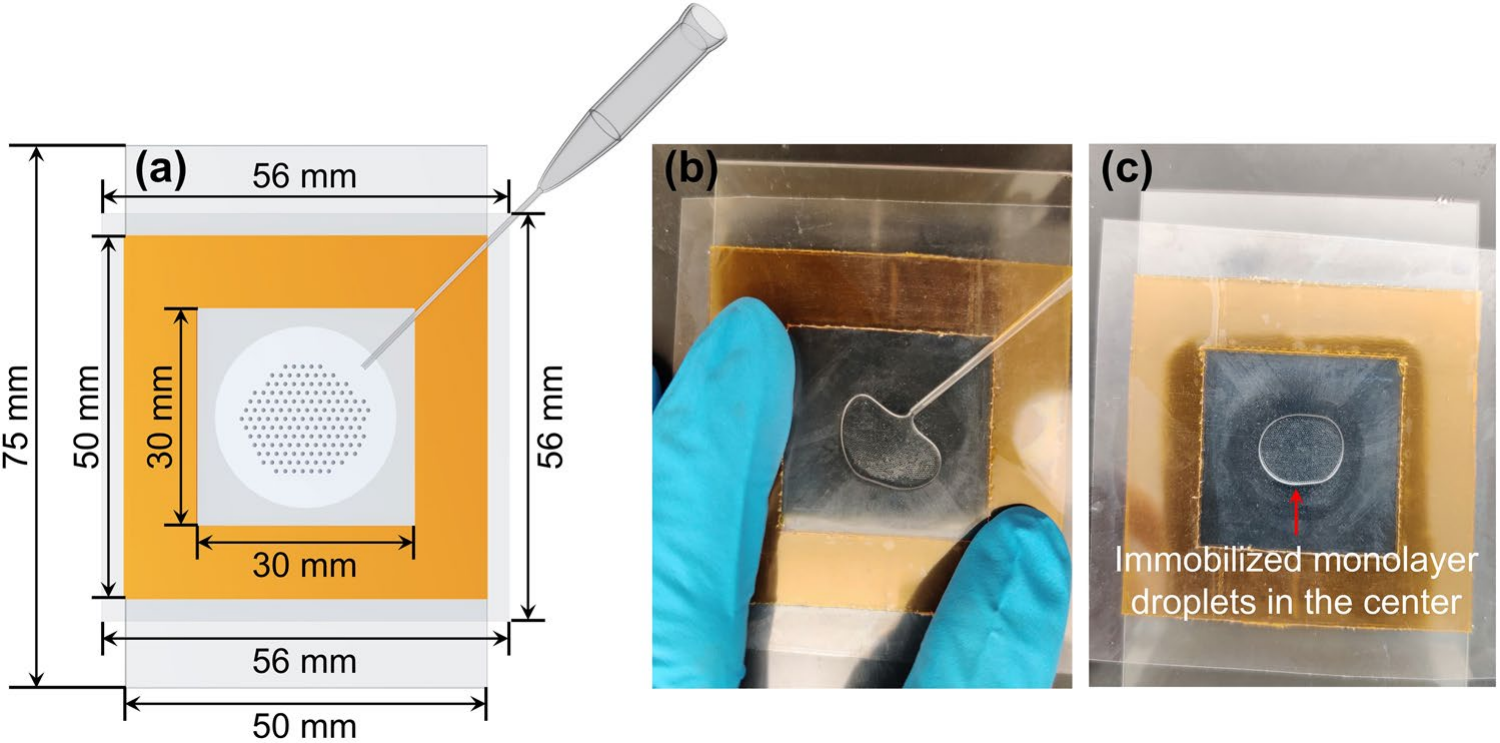
Droplet assembly and imaging chip made of double-sided tape on a glass slide: (**a**) scheme of the chip with a tip at the corner showing how droplets are loaded; (**b**) loading droplets into the chip from a corner by lifting the cover film; (**c**) loaded monolayer droplets immobilized in the shallow center of the chip for subsequent handling and imaging.

The shallow-center design of our assembly chip allows spontaneous assembly at the center of the chip and hence enables manual loading by a pipette (Fig. 5 and Video S2). The droplet assembly is also pinned to the center because of the effect of the sloping channel height on the wetting oil flow, even though they are smaller than the center height. The droplets are hence never in contact with the wall (Fig. 5b,c and Video S2). To minimize contact-line resistance, the wetting contact line will evolve from an irregular shape to an axisymmetric shape around the shallow center after loading the droplet/oil suspension. This contact-line spreading is driven by the wetting oil spreading on the substrate. However, pinning of the suspension droplet to the center is due to the capillary pressure of the concave meniscus at the periphery of the suspension. The wetting oil forms thin films on the substrate to produce a concave meniscus which sustains a negative meniscus oil pressure relative to the surrounding air pressure^43,44^. The magnitude of the capillary pressure is inversely proportional to the channel height at the meniscus. A radially outward protrusion of the suspension will result in a meniscus with a larger radius of curvature at the protrusion tip, due to the larger channel height at that position. This larger meniscus radius results in a less negative meniscus oil pressure and will drain liquid away from the protrusion to arrest the growth of the protrusion. A radially axisymmetric shape at the center is hence approached by the oil suspension—it is pinned to the shallow center. The radial flow during this adjustment towards axisymmetry packs and immobilize the droplets towards the shallow center to form a circular monolayer away from the meniscus periphery. The droplets move toward the center, away from the meniscus at the periphery of the suspension, because of shear-induced migration by the high shear rate at the moving contact line and enhanced hydrophobic repulsion by the substrate there^43^. A circle of assembled droplet monolayer is hence also pinned to the shallow center, concentric to the circular contact line (menisci), even though the droplets are smaller than the gap height at the center. The pinned contact line and droplet monolayer remain stable when the tip is tilted repeatedly, thus allowing transport to the imaging facility. The chamber height of the six-layer tape frame is selected for assembling and immobilizing a single monolayer of pipette generated droplets which simplifies the imaging and analysis of the droplets. Dimensions of the chip can be changed to accommodate droplets of different sizes and amounts. As long as oil wets the chip that has a chamber with a shallow center, droplets will be pinned at the shallow center of the chip chamber to simplify the imaging and handling of droplets. In addition, a notch could be cut at a corner of the tape frame so that pipette tips can be inserted to the chip chamber center for droplet loading without lifting the covering film (Fig. S6). In terms of droplet unloading from the chip, it is just a reversed process of droplet loading (Video S3).

**Figure 6:**
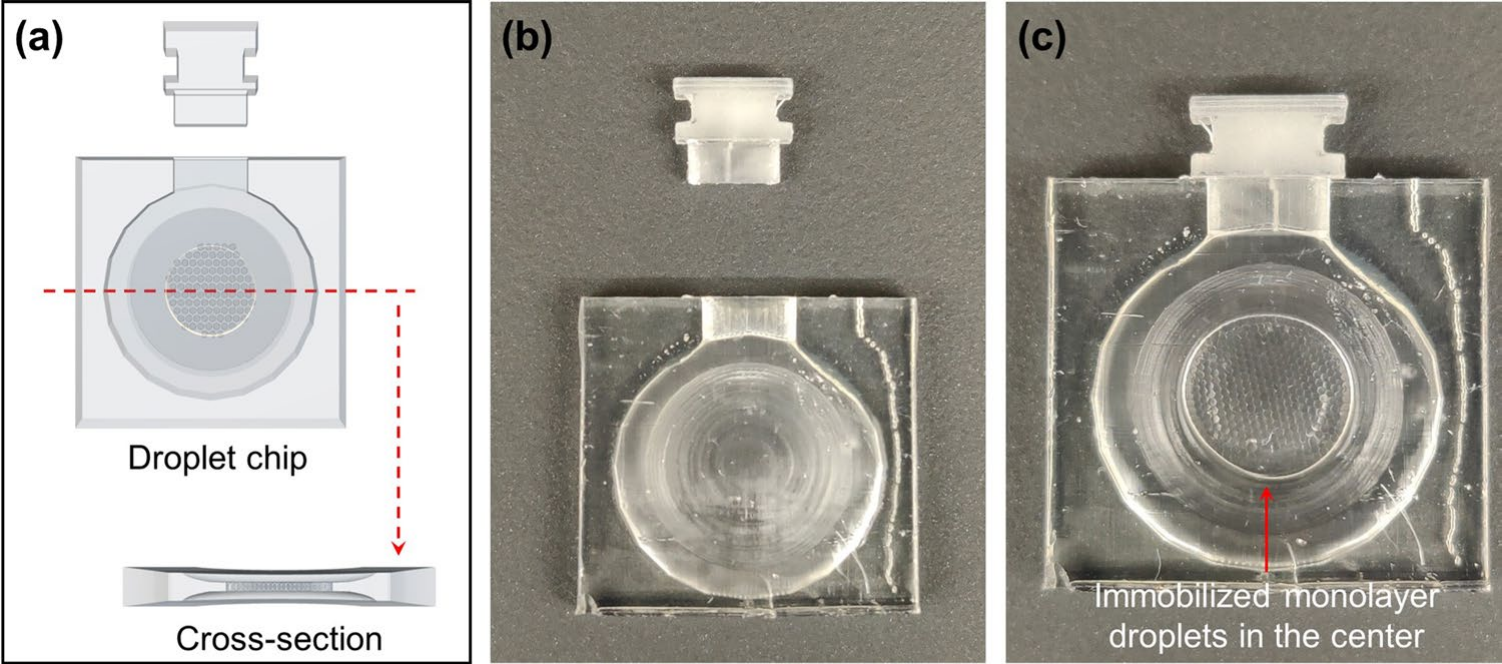
A 3D printed droplet assembly and imaging chip with matched plug: (**a**) the 3D model of the chip and the cross-section to show the shallow center design; (**b**) a printed chip; (**c**) monolayer droplets immobilized inside the chip for subsequent handling and imaging. The droplets will stay still away from the walls inside the chip because of the sloping interior chamber height towards the center. The exterior dimension of the droplet imaging chip is 20 mm × 20 mm × 2.5 mm.

The shallow-center chip can also be fabricated by 3D printing with a clear resin that oil wets (Fig. 6). The chip is designed to have a sloping height from the wall to the center, which also has a channel height of about 600 microns as for the tape-based chip. As expected, assembled droplets remain immobilized in the center during handling and imaging (Fig. 6c). One issue with this resin 3D printed chip is that the chip blocks green fluorescence, possibly because of the initiator residues. This problem can be solved if injection molding is applied to fabricate the chip from melted polymers.

### Example application I: droplet digital polymerase chain reaction (ddPCR) for quantifying low concentration target RNAs using droplets as microreactors

With a sufficient number of nanoliter microdroplets, ddPCR detection and quantification of single nucleic acids is attainable. As a general example, human GAPDH PCR reagent is used to detect and quantify human RNA. Herein, RNA rather than DNA is chosen as the target so that reverse transcription is also conducted. The human GAPDH PCR reagent kit is broadly applied in bioengineering/biology labs as a basic endogenous control for the normalization of other sample nucleic acids in bulk real-time quantitative polymerase chain reaction tests^45,46^. The inexpensive GAPDH PCR reagent kit and human control RNAs are hence ideal for training purposes. Droplets with PCR reaction mix will flocculate to the top of the denser fluorinated oil in regular PCR tubes and tend to coalesce during thermal cycling. The problem can be solved by adding ~ 1 w/v% F127 to the aqueous phase as a co-surfactant.

**Figure 7:**
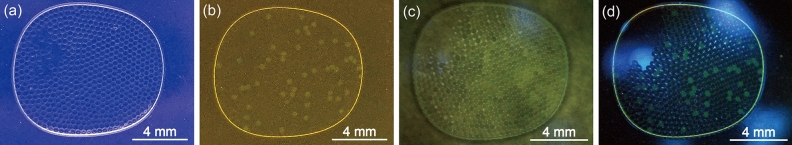
Imaging ddPCR droplets by smartphone together with transilluminator without (**a**) or with (**b**) fluorescence illuminating/filtering and by handheld mini microscope without (**c**) or with (**d**) fluorescence illuminating/filtering after 40 thermal cycles. The droplets are generated via a pipette tip deformed by 20 inch-pound torque force. Both methods are adequate to image the droplets properly.

For the fluorescence imaging of droplets after ddPCR, two approaches are suggested for this kit. The first is to take advantage of a common smartphone camera and the transilluminator that is used frequently in labs for viewing electrophoresis gels. Because the droplets generated by pipetting are usually several hundred microns in size, they are large enough to be observed visually and imaged by smartphone cameras. With blue light illumination and amber filter filtering from the transilluminator, green fluorescence (FAM dye) of droplets can be recorded (Fig. 7b). If the smartphone and transilluminator combination is not practical, a second approach is to use an inexpensive handheld fluorescence mini microscope (Fig. 7d). Both methods have been tested after PCR reaction and the positive droplets (droplets with targets as indicated by the amplified strong green fluorescence) are clearly observable in both cases (Fig. 7). Though the droplet fluorescence image taken by the handheld mini microscope has better contrast (Fig. 7d) than that taken by smartphone camera (Fig. 7b), the number of discernible positive droplets are the same for images from the two approaches. Therefore, reliable and accessible solutions are available for imaging droplet fluorescence after ddPCR reaction.

**Figure 8:**
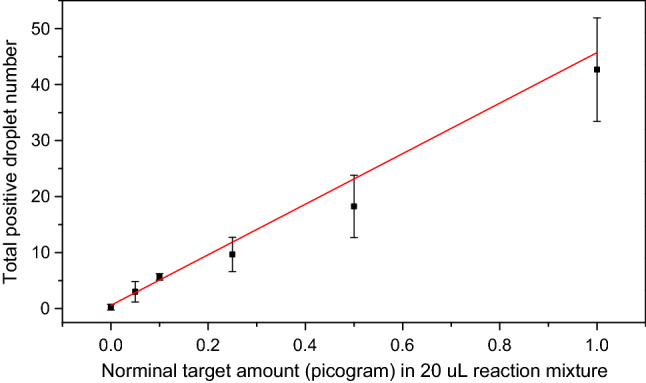
Positive droplet counting based quantification of low target concentration samples. Error bars are standard deviations.

The effective droplet fluorescence imaging leads to the quantification of target nucleic acids. For training and prototyping purposes, quantification is best done by counting the positive droplets, without knowing the total number of droplets or invoking Poisson statistics. At low copy numbers, when the droplets significantly outnumber the targets, the total number of positive droplets is roughly equal to the total number of targets. Positive droplet quantification requires negligible sample loss during droplet generation and imaging. The pipetted nanoliter droplets meets all the requirements for counting-based quantification of low target concentration samples. Repeated experiments are conducted for several low target concentration (0–1 picogram in 20 µL reaction mixture) samples and the positive-droplet-counting-based quantification results are shown in Table 1 and Fig. 8. The linear relationship indicates that the method is sufficient for rough quantification and accurate detection for low target concentration samples (Fig. 8). Though the standard deviations increase as the target concentrations increase, the coefficient of variations soon becomes stable in the 20–30% range (Table 1) and the variations are probably caused by target concentration fluctuation in the low concentration sample solution, volume variation of the micro pipettor, and target molecule affinity to the PCR tube and pipette tip surfaces. In addition, the calculated limit of detection^47^ based on average false positive, standard deviation of blank samples, and standard deviation of lowest non-negative concentration samples from Table 1 (LoD = 0.2 + 1.645*0.45 + 1.645*1.82 = 3.92) is 3.92 copy per 20 µL reaction mix which is comparable to commercial ddPCR products and indicates the pipette ddPCR is good for accurate detection despite the counting-based quantification is more like rough estimation due to the 20–30% variations. (Table 1) Though human RNA is used in this demonstration, this technique can potentially be applied to quantify virus RNAs and reduce the false negative detection rate of PCR tests for SARS-CoV-2 viruses in heterogeneous specimens^48,49^. Consequently, the kit offers good training of droplet digital PCR for nucleic acids detection and quantification.

**Table 1:** Repeated droplet digital PCR experiments with low target concentration samples.

Nominal target amount (picogram) in 20 µL reaction mixture	Total positive droplet number					Average number	Standard deviation	Coefficient of variation (CV) (%)
	Exp. 1	Exp. 2	Exp. 3	Exp. 4	Exp. 5			
0	0	1	0	0	0	0.2	0.45	223.6
0.05	1	4	2	5	1	2.6	1.82	69.9
0.1	6	5	6	3	6	5.2	1.30	25.1
0.25	13	9	7	9	11	9.8	2.28	23.3
0.5	16	26	18	13	22	19	5.10	26.8
1	32	48	48	55	38	44.2	9.12	20.6

### Example application II: droplet encapsulation and culturing of microorganism spirulina using droplets as microcompartments

**Figure 9:**
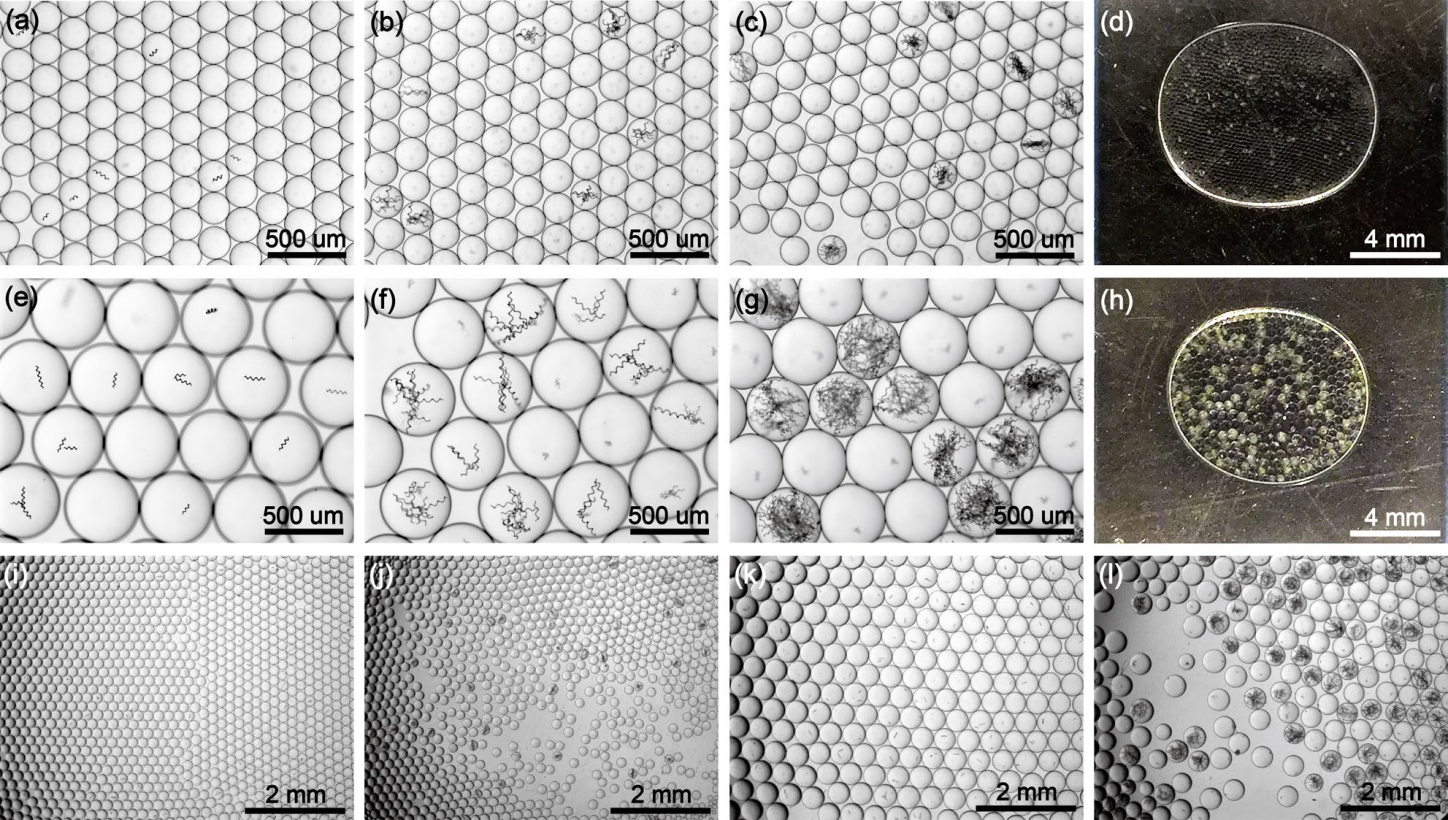
Photosynthetic culturing of spirulina in small (**a**–**d**) and large (**e**–**h**) microdroplets inside tape-based imaging chips: (**a**,**e**) spirulina in as-generated droplets; (**b**,**f**) proliferated spirulina in positive droplets after 5 days of culturing; (**c**,**g**) proliferated spirulina in positive droplets after 10 days of culturing; (**d**,**h**) smartphone imaging of the 10-days culturing droplets showing recognizable color/turbidity differences of positive droplets with spirulina especially in large microdroplets; (**i**–**l**) low magnification images corresponding to (**a**,**c**,**e**,**g**) respectively.

Microdroplets provide isolated microcompartments and microenvironments for cells or microorganisms to inhabit and this feature is actively used for single cell/microorganism analysis/screening^22–25^ and also for emerging droplet/cell factories^50,51^. For many of these applications, the droplets offer indispensable manipulation and assay of single cells or microorganisms. As an example, to show droplet encapsulation with our proposed kit, single microorganism spirulina is cultured in small and large microdroplets inside tape-based imaging chips (Fig. 9) or PCR tubes (Fig. S7). Spirulina is a type of edible cyanobacteria with outstanding photosynthetic efficiency that produces a variety of nutrients and ingredients for animals and humans. The photosynthetic growth of spirulina is vigorous because the high O_2_/CO_2_ solubility (10–20 × higher than water) in fluorinated oil sustains microorganism proliferation^52,53^. In time, the spirulina grows in number and eventually occupies the entire droplet (Fig. 9, S7). After 10 days of culturing, the calculated proliferation rate (number of droplets with at least four copies of spirulina / total number of droplets with spirulina) is 96.9 ± 1.8% from six times of experiments. The growth of spirulina in smaller microdroplets are relatively slower, probably because of the more limited space and nutrient supply. (Fig. 9a–d, i, j) The confinement of droplets is effective, and no spirulina is observed to diffuse or transfer into other droplets (Fig. 9c,g,j,l). Moreover, the confinement of droplets isolates and protects the spirulina by the virtue/soft walls (droplet/oil interfaces) formed with biocompatible surfactants^54^. Though the droplets may shrink after days of photosynthetic growth due to water evaporation, the pipette generated large microdroplets are still large enough for smartphone imaging and the continued growth means spirulina microorganisms are alive for over 10 days (Fig. 9e–h, S7). The color or turbidity differences caused by the proliferated spirulina in positive droplets confirm successful culturing and imply digital quantification of low concentration microorganism samples after proper culturing, such as those reported for bacteria droplet digital quantification^55^.

### Example application III: controlled synthesis of capped polyacrylamide/gold composite microgels using droplets as templates/molds

**Figure 10:**
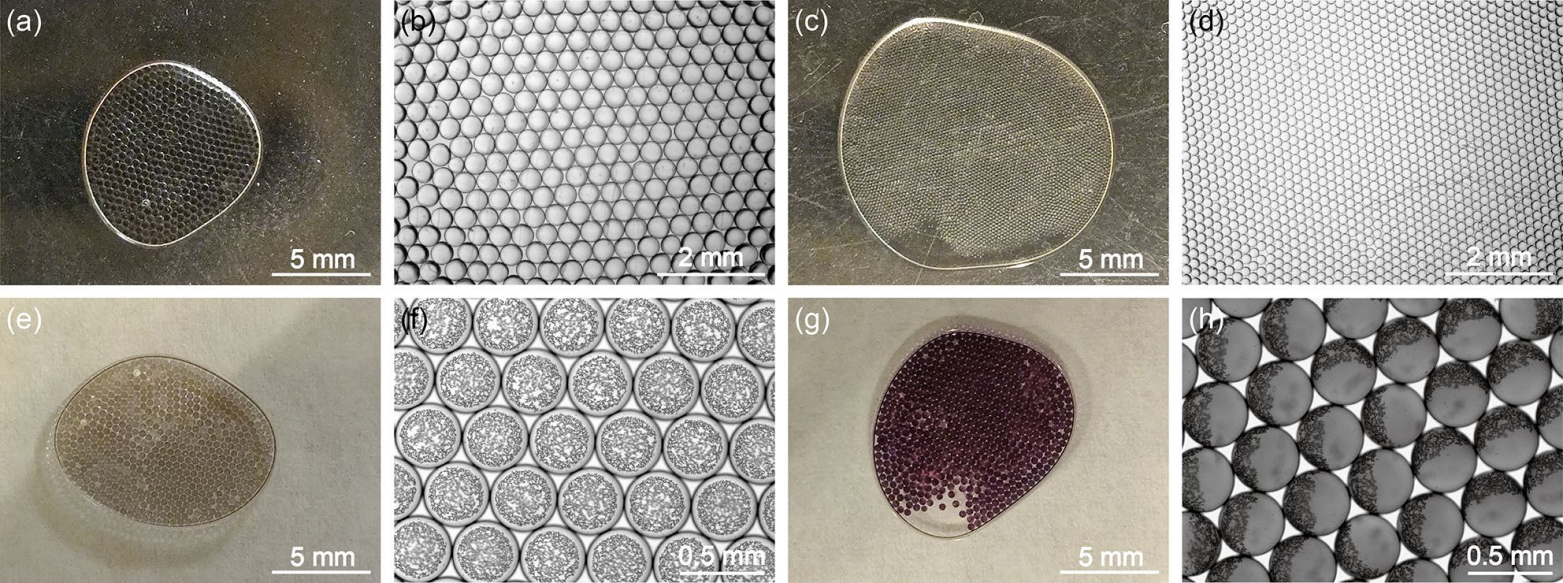
Controlled synthesis of microgels: (**a**–**d**) Smartphone (**a**,**c**) and microscope (**b**,**d**) images of two sizes of polyacrylamide microgels prepared from 20 µL mixtures with different pipette tips. (**e**,**f**) Droplets with gelation precursors and gold reduction sources before gelation. In the liquid phase droplets, the reduced gold will precipitate to the bottom of the droplets because of its higher density. The droplets are UV crosslinked to synthesize the capped polyacrylamide/gold composite microgels in (**g**,**h**). The UV gelation process will also expedite gold reduction and the in situ formed polyacrylamide network confines the growth of reduced gold to produce (**g**) wine red microgels filled with gold nanoparticles. Previously precipitated gold at the bottom of each liquid droplet will be fixed in place to eventually prepare (**h**) microgels with gold caps. Images of (**a**,**c**,**e**,**g**) are taken by smartphone while images of (**b**,**d**,**f**,**h**) are taken by a table-top microscope.

Intuitively, droplets are excellent templates/molds for microgel synthesis. With the initiation of crosslinking, the liquid droplets with acrylamide polymerization precursors are turned into uniform microgels (Fig. 10a–d). The liquid to gel transformation in droplets is further utilized to synthesize polyacrylamide/gold composite microgels with gold caps (Fig. 10e–h). The idea is to exploit the different kinetic time scales of the gelation/precipitation reactions and the different physical properties of their products to pattern the synthesized materials. Generally, the gel network has a confinement effect on the interior synthesis reaction which not only prevents sedimentation of precipitates but also introduces surface adsorption/reaction to produce materials with distinct structures/morphologies. For example, different crystal structures in bulk liquid and gel network have been utilized to synthesize patterned single crystals by programmable incorporation of foreign materials^56^. Herein, the acrylamide gelation precursor is mixed with 100 mM sodium citrate and 50 mM gold (III) chloride and this solution will be stable for about 6 min before noticeable gold reduction happens. The 6-min time period is sufficient for pipette droplet generation of the 20 µL mix which usually takes about 3 min to complete. Gold reduction in as-generated liquid phase droplets is fast and a good amount of reduced gold precipitates to the bottom of each droplet in about 10 min after droplet generation (Fig. 10e,f). With UV exposure for 2 min, liquid droplets are polymerized and crosslinked into microgels, while trapping the earlier precipitated gold at the bottom hemisphere to form gold caps on the microgels (Fig. 10g,h). After the disturbances (rotation and movement) caused by subsequent manipulations for imaging, the gold caps are randomly oriented (Fig. 10h) which indicates the formation of microgels because otherwise the gold gaps will all precipitate to the bottom like in the pre-crosslinking liquid droplets (Fig. 10f). Moreover, perhaps because of the confinement and gold reducing effects from the in situ polyacrylamide network formation^57^, the newly reduced gold immediately fuse into gold nanoparticles in the gel-network nanopores of the microgel upon gelation, as indicated by the classical wine-red color of the microgels (Fig. 10g). This example of controlled synthesis of capped polyacrylamide/gold composite microgels indicates that droplets are promising molds and templates for synthesis of functional patterned microgels or particles with desired structures and compositions^33,58^.

## Conclusion

In summary, this work has provided a homemade rapid prototyping kit for droplet microfluidics that can be used by beginners and non-expert researchers from diverse backgrounds. The challenging and tricky droplet microfluidics workflow of droplet generation/assembly/imaging, often requiring expensive commercial equipment that is difficult to tune, is simplified by pipette droplet generation with easily fabricated elliptical pipette tips, droplet assembly and immobilization in the shallow-center chip (fabricated with tapes or 3D printing), and droplet imaging by smartphone or portable mini microscope. These designs allow easy generation of uniform droplets with varying sizes and with a dispersed fluid of arbitrary viscosity, surface tension and cellular content. Three examples are chosen and conducted to demonstrate the versatile applications of this droplet microfluidic platform on single molecules, single microorganisms and material synthesis with droplets used as microreactors, microcompartments and templates/molds, respectively: (i) droplet digital PCR reaction in droplets to quantify low copy RNAs at the point of need without purchasing expensive instruments; (ii) spirulina photosynthetic culturing in droplets; (iii) controlled synthesis of capped polyacrylamide/gold composite microgels. Though the three examples are not pursued in-depth, we have demonstrated that the kit can be used to support extensive rapid prototyping and testing. Therefore, the pipette droplet kit should facilitate the design, prototyping and personnel training of droplet microfluidic technologies by non-expert users.

## Methods

### Elliptical pipette tip preparation

To standardize the tip modification process, a torque-screwdriver-based tool is assembled. Basically, the tool transfers the torque forces into pressures to deform the pipette tips. A metal clamp is combined with a 3D printed case which will prevent the rotation of glass/metal slides and the movement of pipette tips while deforming. A model file of the 3D printed case is provided in Supporting Information.

For the preparation of an elliptical pipette tip, the head part of a regular round orifice tip (Fisherbrand™ Gel-Loading Tips, 1–200μL, default tips used in this work if not other specified) is inserted in between or under the two glass/metal slides mounted on the tip modification tool. The channel on the 3D printed case help locate the tips and the interval barriers on the channel walls help determine how long the tip is inserted for deformation so that the tips are deformed in a more controllable and reproducible way. A certain torque force is directly set on the torque screwdriver and the screwdriver is used to drive the screw on the metal clamp until the desired torque is reached. After ten seconds, the torque is released and the deformed tip is removed. While the screwdriver is compressing the tip, the glass/metal slides cannot rotate freely in the 3D-printed case and hence transform the torque to pressure to deform the head part of the pipette tip to the desired elliptical cross-section. The rigid glass slides will break when the torque force is over 20 inch-pound, and stronger metal or other material slides/plates can be used to deform tips under higher deformation torque forces.

For the measurement of tip orifice aspect ratios, ~ 2 mm long pieces from the orifice are cut off the as-bought or deformed tips by new razor blades and the cut pieces are adhered vertically onto double-sided polyimide Kapton tape with one tape side already attached to a regular glass slide. For easy imaging purpose, the original tip orifice surfaces are in contact with the tape surface. Afterwards, the tip orifices on the glass slide are imaged by microscope (Olympus IX71) and the two axes (long & short) of the orifices are measured by two orthogonal coordination line segments in Adobe Illustrator (Fig. S8). Briefly, for a tip orifice image, a line is drawn to indicate the long axis and then the line is copied in place and rotated 90°. The copied and rotated line is adjusted to the length of the short axis of the orifice (Only the length is changed, no rotation or offset of the line so that the long and short axis lines are perpendicular to each other and intersect at the middle point of the long axis). Finally, the lengths of the two lines are recorded and used to calculate the aspect ratio, namely (long axis length)/(short axis length).

### Pipette droplet generation

The head-flattened pipette tip is attached to a regular 20 µL micro pipettor. Afterward, ~ 10 µL of fluorinated oil (HFE 7500 with 2 wt % RAN-008 surfactant, RAN Biotechnologies) is aspirated into the pipette tip and subsequently pipetted out of the tip into a 200 µL PCR tube (Cole-Parmer, item # EW-67103-90) pre-loaded with 40–50 µL fluorinated oil. The pipette tip is air dried for about 50 s. The micro pipettor with the deformed tip is then used to aspirate the sample liquid. The pipette tip with sample liquid is inserted into the pre-loaded 40–50 µL fluorinated oil in the 200 µL PCR tube. After a few seconds, the oil will wet inside and around the tip orifice surface. For some cases (like with viscous sample liquids, highly deformed tips etc.) oil does not wet inside the tip head much, backward adjusting the micro pipettor to increase volume by 0.5–1 µL would be helpful to drive the oil into the tip head and wet the tip inner surfaces. After wetting the tip head with oil, the sample liquid inside the tip is slowly pipetted out into the oil to achieve an automatic pinch-off into uniform droplets. The recommended pipetting flow rate is less than 10 µL/min. Since droplet generation is not sensitive to small flow rate changes, manual pipetting to achieve droplet generation is possible for high aspect ratio elliptical tips. An electric micropipettor with a controlled flow rate is recommended, however, for automated pipetting droplet generation.

### Droplet assembly and imaging chip fabrication

Our unique shallow-center chip is made of six layers of common double-sided polyimide tape on a glass slide and the top is covered by a piece of the tape protection film (tape liner) after cutting a chamber out of the layered tape for droplets loading. When placing the tape protection film (the film side originally in contact with the tape is faced down and later attached to the tape frame), the clear film at the center of the tape frame is pressed onto the glass slide surface simply by thumb and then the skirt of the clear film is adhered to the tape frame tightly. Because the clear film at the center is pressed down during covering, though the film will bounce back a little after pressure release, the gap between the film and the glass slide becomes narrower at the center of the tape chip (Fig. S9). It is recommended to press the film hard both the center to the glass slide surface and the skirt to the tape frame to make the center gap as narrow as possible especially for not that adhesive tape frames so that a shallow center is there even after conner lifting up during sample loading. Once there is a shallow center, the very accurate control of the narrow gap height is not necessary. Before loading samples, pure oil phase could be used to test the chip to make sure the shallow center could stabilize the oil in the center well. If the oil phase floats away to the tape frame walls, remove the oil and redo the film covering/attaching step or make a new chip if the tape frame is no longer adhesive. The dimension of the chip is quite flexible and a chip with a 75 mm by 50 mm glass slide, an exterior 50 mm by 50 mm and interior 30 mm by 30 mm tape frame and a 56 mm by 56 mm cover film is recommended for initial trials (Fig. 5a). As mentioned, the gap height at the center for six-layer tape-based chip is roughly about 600 microns which can be roughly estimated by outer diameters (OD) of certain pipette tips like the 0.57 mm OD tip used for droplet loading in this work. Some adjustment of the reduced center height of the chamber by using less layers of tape may be necessary for smaller droplets. The chip is reusable until the tape frame is no longer sufficiently adhesive to maintain a shallow center chamber structure. Super adhesive double-sided tape is recommended for more durable chips. Two layers of expensive PCR frame seal chamber (Bio-Rad, 15 × 15 mm, 65 µL #SLF0601) could be used to make the imaging chip instead if cutting a chamber out of the six-layered polyimide tape is a problem.

The shallow-center assembly chip can also be 3D printed instead of fabrication by the above adhesive tape method. The chip is designed by a free online software (Tinkercad) and printed by a cheap popular resin 3D printer (ELEGOO Mars 2 Pro) with a clear resin (Siraya Tech Blu 3D Printer Resin Clear V2). The 3D model is sliced by a free software (CHITUBOX Basic) with 4 s exposure time, 40 s bottom exposure time, lift distance 8 mm, 50 µm printing layer height, lift speed 80 mm/min, and retract speed 210 mm/min. After printing, the chip is washed with 25–50% resin solution in isopropyl alcohol (IPA). Pure IPA wash is not recommended because it will over-clean the incompletely crosslinked chip and make the chip surfaces rough and less transparent. The clean chip is UV-cured for four times at 5-min exposure in a curing box (ELEGOO Mercury Plus 2 in 1 Washing and Curing Station V2.0) before usage.

### Droplet assembly and imaging in chips

An as-bought round orifice pipette tip (VWR^®^ Gel-Loading Pipet Tips, 1–200 µL, Round tip, 0.57 mm Outer Diameter) is used to aspirate the droplet/oil suspension from the container. The pipette then delivers the suspension to the center of the imaging chip through one corner of the adhesive-film chip by slightly lifting the cover film at that corner (see video in Supporting Information). Alternatively, a notch can be cut out of the tape frame at a corner for inserting the tip through the notch to the center of the imaging chip without lifting the cover film. A pipette inlet is fabricated for the 3D printed chip for the same purpose. Importantly, because of the shallow center structure (reduced height), the suspension will not flow freely despite the wetting oil. Instead, it will be pinned at the center of the imaging chip for convenient optical imaging by a smartphone (OnePlus 7 Pro) or a microscope (Olympus IX71). Microscope images are used for droplet size and uniformity analysis to validate the pipette droplet generation performances. Briefly, a scale is set in ImageJ according to the microscope scale bar and then the projection areas of individua droplets are measured to calculate the radius of each droplet. Because the height of the imaging chip chamber is about 600 µm, droplets larger than that may flatten a little when loaded and assembled inside the imaging chip, thus producing increased projection areas and subsequently increased radii than normal.

For fluorescence imaging, the chip with droplets is placed onto a blue light transilluminator (Clare Chemical Research) and then an amber filter is put on top of the imaging chip. The fluorescence of droplets is imaged with a smartphone in dim or dark circumstances after tuning on the transilluminator. If a transilluminator is not available, an affordable handheld mini fluorescence microscope (Dino-Lite AM4115T-GFBW) is recommended for droplet imaging.

### Droplet digital polymerase chain reaction

A typical reaction mix is prepared with 5 µL Luna Probe One-Step RT-qPCR 4X Mix (New England Biolabs, Catalog # M3019S), 1 µL human GAPDH primers and probes (Thermo Fisher Scientific, Catalog # 4333764 T), 2 µL 10 w/v% F127 aqueous solution, 0–10 µL diluted human control RNA (Thermo Fisher Scientific, Catalog # 4,307,281) as target and 12–2 µL nuclease-free water to make the total mix volume of 20 µL. The frozen reagents are melted on ice and mixed by pipetting before usage and are returned to the refrigerator immediately after usage. The 10 w/v% F127 aqueous solution is prepared by dissolving 0.1 g of F127 powder (Sigma-Aldrich, BioReagent, Catalog # P2443-250G) into 1 mL cold nuclease-free water (Thermo Fisher Scientific, Catalog # R0582) and the solution is stored at 4 °C. Once the reaction mix is completed, uniform droplets are generated in PCR tubes as mentioned previously. The PCR tube with reaction mix droplets is immediately placed into a portable thermal cycler (Bio-Rad, MJ Mini Thermal Cycler) for PCR reaction with a protocol of 10 min reverse transcription at 52 °C, 2 min denaturation at 95 °C, 40 cycles of 10 s at 95 °C and 30 s at 60 °C.

### Spirulina culturing in droplets

The spirulina (ACAp-01003) and corresponding complete culture media kit (salts + nutrients, MKAp-00001) are from Algae Research Supply. The culture media is prepared by dissolving the salts and nutrients in a certain amount of water recommended by the supplier. The spirulina suspension is then diluted with the culturing media to desired concentrations. The well mixed spirulina and culturing media are dispersed into droplets by pipetting droplet generation with an elliptical tip deformed by 20 inch-pound torque force. After encapsulating the microorganism, the droplets (loaded in double-sided-tape-based imaging chips or in 200 µL PCR tubes, together with the droplet generation oil) are placed on a windowsill with sunlight for spirulina growth and proliferation. The corner of the imaging chip and the cap of the PCR tube are opened once a day for about 1 min for gas exchange purpose during spirulina culturing in droplets. Under these conditions, the life cycle of spirulina is 2–3 weeks.

### Controlled microgel synthesis

For polyacrylamide microgel preparation, 5 µL of monomer and crosslinker solution (Acrylamide: Bis-Acrylamide 29:1, 40% Solution, Fisher BioReagents, BP1408-1) is mixed with 13 µL deionized water and 2 µL 2 w/v% initiator solution. The 2 w/v% initiator solution is prepared by dissolving 10 mg Lithium phenyl-2,4,6-trimethylbenzoylphosphinate (LAP, Sigma-Aldrich, 900,889-1G) powder into 500 µL water and the solution containing tube are wrapped by an aluminum foil and placed in the dark when not in use. The resulting 20 µL precursor mix is generated into droplets by pipetting with an elliptical tip deformed by 20 inch-pound torque force. Besides this pipette tip, another type of tip (Eppendorf microloader tip) with reduced orifice size is also deformed by the same force and used to generate droplets with smaller sizes. The generated droplets are transformed into uniform microgels after 2 min of irradiation in a UV box (Electro-Lite, Electro-Cure 500).

For the controlled synthesis of polyacrylamide/gold composite microgels with gold caps, the solution is prepared with 10 µL water, 5 µL of monomer and crosslinker solution (Acrylamide: Bis-Acrylamide 29:1, 40% Solution, Fisher BioReagents, BP1408-1), 2 µL 2 w/v% LAP initiator solution, 2 µL 1 M sodium citrate (Sigma-Aldrich, W302600-1 KG-K) solution and 1 µL 1 M gold chloride (Sigma-Aldrich, 520,918-1G) solution. After mixing well, the solution is immediately generated into droplets by pipetting with an elliptical tip deformed by 20 inch-pound torque force. The newly reduced gold will precipitate to the bottom of each droplet. Ten minutes later, the droplets are crosslinked to form microgels by placing them in a UV box for 2 min. The gelation process will affix the precipitated gold in place to form caps on the uniform microgels.

## Supplementary Information

Supplementary Information 1.

Supplementary Information 2.

Supplementary Information 3.

Supplementary Information 4.

Supplementary Video 1.

Supplementary Video 2.

Supplementary Video 3.

## Data Availability

The authors declare that all data supporting the findings of this study are available within the paper and the Supplementary Information.
