# Supplementary material for: A home-made pipette droplet microfluidics rapid prototyping and training kit for digital PCR, microorganism/cell encapsulation and controlled microgel synthesis
Source: Sci Rep. 2023 Jan 5;13:184. doi: 10.1038/s41598-023-27470-1 (PMC9813469; doi:10.1038/s41598-023-27470-1)
Supplement: Supplementary file 4 — Supplementary Information 4. [file 41598_2023_27470_MOESM4_ESM.docx]

**Supporting Information**

**A Home-Made Pipette Droplet Microfluidics Rapid Prototyping and Training Kit for Digital PCR, Microorganism/Cell Encapsulation and Controlled Microgel Synthesis**

Liao Chen^†^, Chenguang Zhang^†^, Vivek Yadav^†^, Angela Wong^†^, Satyajyoti Senapati^†^, and Hsueh-Chia Chang^†⁎^

† Department of Chemical and Biomolecular Engineering, University of Notre Dame, Notre Dame, Indiana 46556, United States

⁎ Corresponding author: hchang@nd.edu


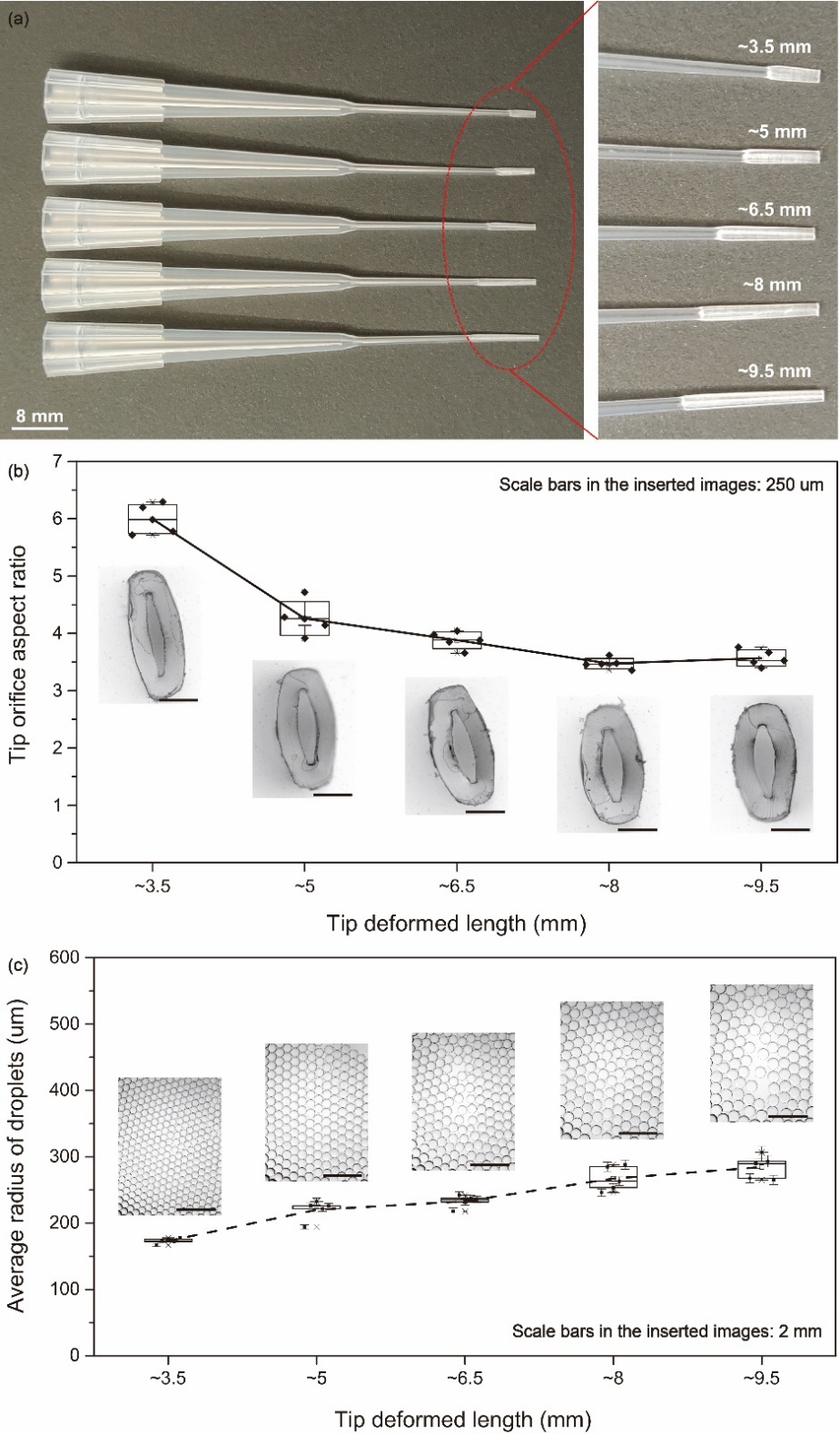


Figure S1. Controlled pipette tip modification by changing deformed lengths under a constant (20 inch pound) torque force and corresponding pipette droplet generation: (a) camera images of deformed tips with various deformation lengths; (b) cross-section aspect ratios of pipette tips versus deformed lengths, inserted images are typical deformed tip orifice cross-sections; (c) average radii of water droplets generated with deformed pipette tips, each point represents average radius and standard deviation (error bar) of droplets generated by a deformed tip and the inserted images are droplets generated by the tips with orifice images inserted in (b).


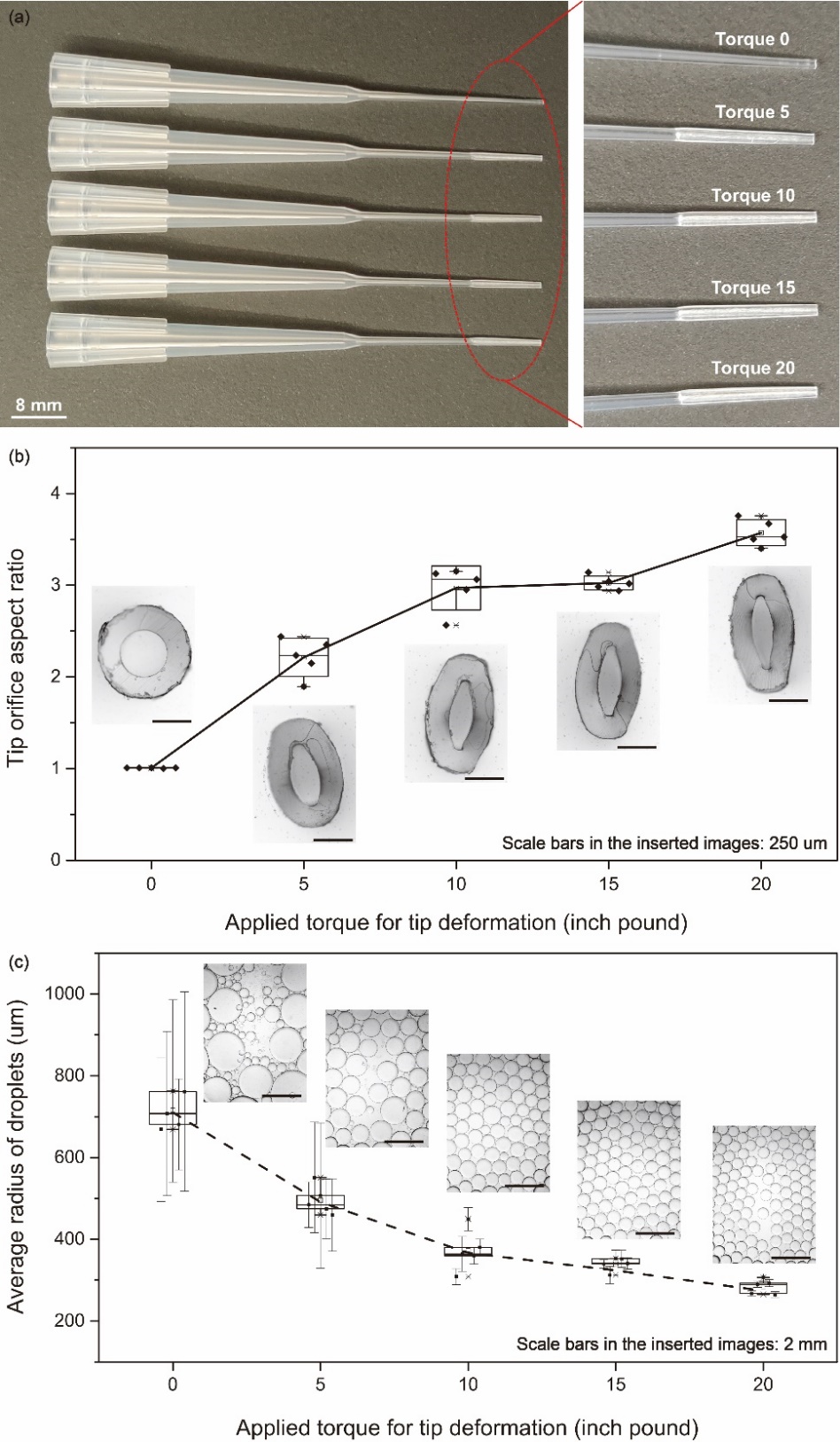


Figure S2. Controlled pipette tip modification by changing torque forces with constant deformed length (~9.5 mm) and corresponding pipette droplet generation: (a) camera images of deformed tips under various torque forces; (b) cross-section aspect ratios of pipette tips versus applied torque forces, inserted images are typical deformed tip orifice cross-sections; (c) average radii of water droplets generated with torque deformed pipette tips, each point represents average radius and standard deviation (error bar) of droplets generated by a deformed tip and the inserted images are droplets generated by the tips with orifice cross-section images inserted in (b).


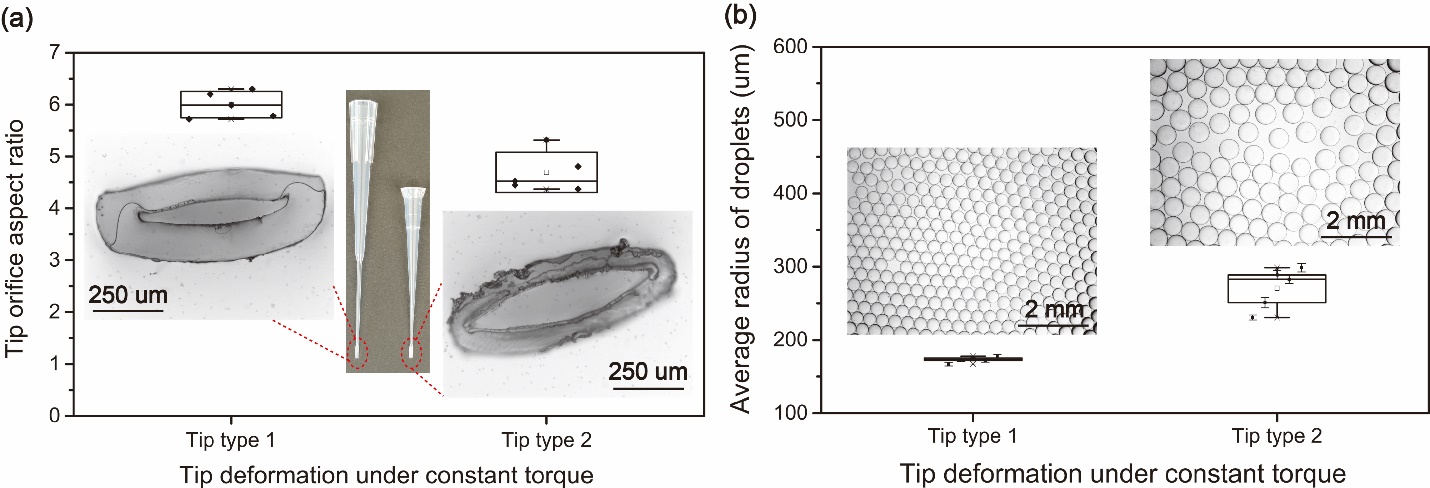


Figure S3. Two types of tips deformed by same torque force (20 inch pound) with same deformed length (~3.5 mm): (a) orifice cross-section aspect ratios of deformed 200 µL tips (Tip type 1, the default tip type used in his work) and 10 µL tips (Tip type 2), inserted images are camera images (insert, middle) of the two types of tips (insert, left & right) and the corresponding deformed tip orifice cross-sections; (b) average radii of water droplets generated with the two types of torque deformed pipette tips, each point represents average radius and standard deviation (error bar) of droplets generated by a deformed tip and the inserted images are droplets generated by the tips with orifice cross-section images inserted in (a).


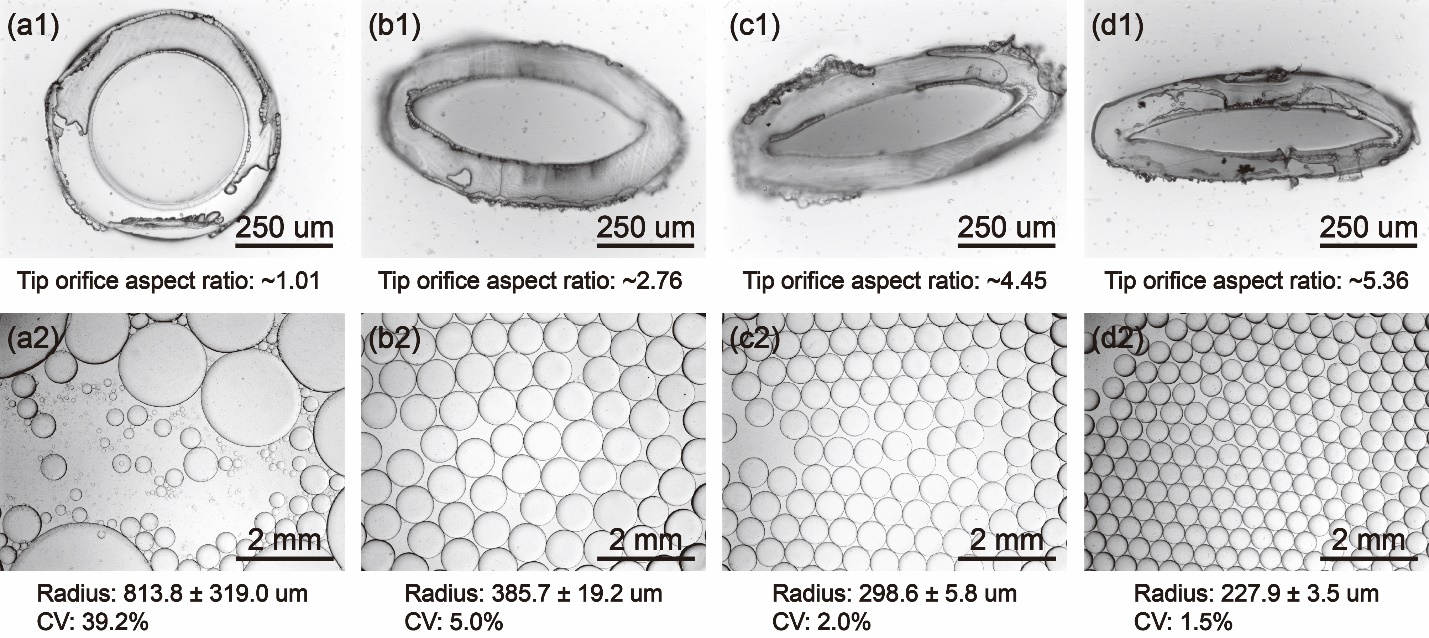


Figure S4. The tip orifices and the generated water droplets corresponding to the 10 µL tips deformed with 0 (a1, a2), 10 (b1, b2), 20 (c1, c2), 30 (d1, d2) inch pound torque forces. Tip deformation helps generate smaller and more uniform droplets.


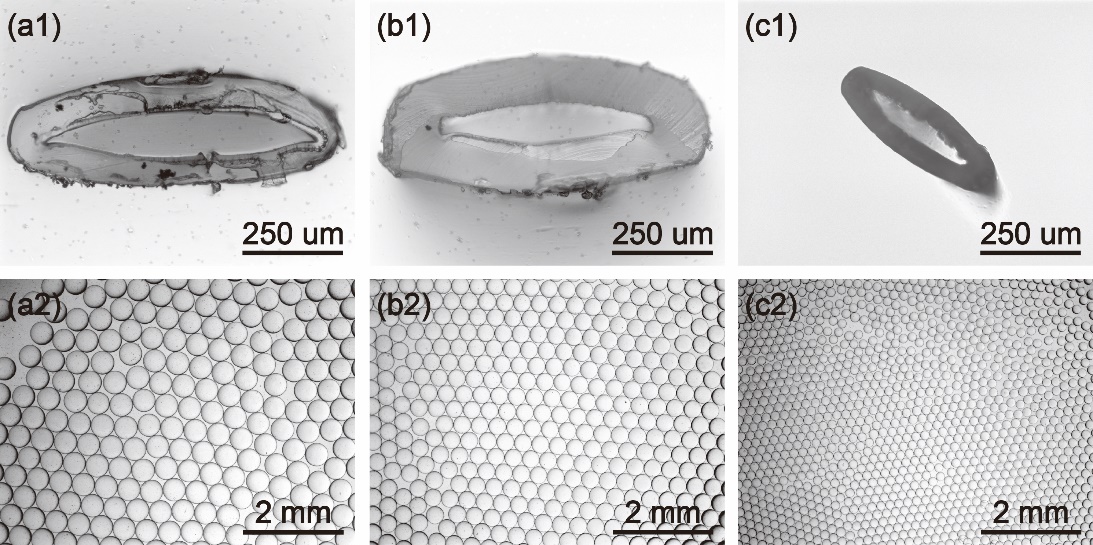


Figure S5. Pipette droplet generation with deformed tips of similar orifice aspect ratio (~5.4) but different orifice sizes. When aspect ratio is similar, the smaller orifice size tip helps generate smaller droplets.


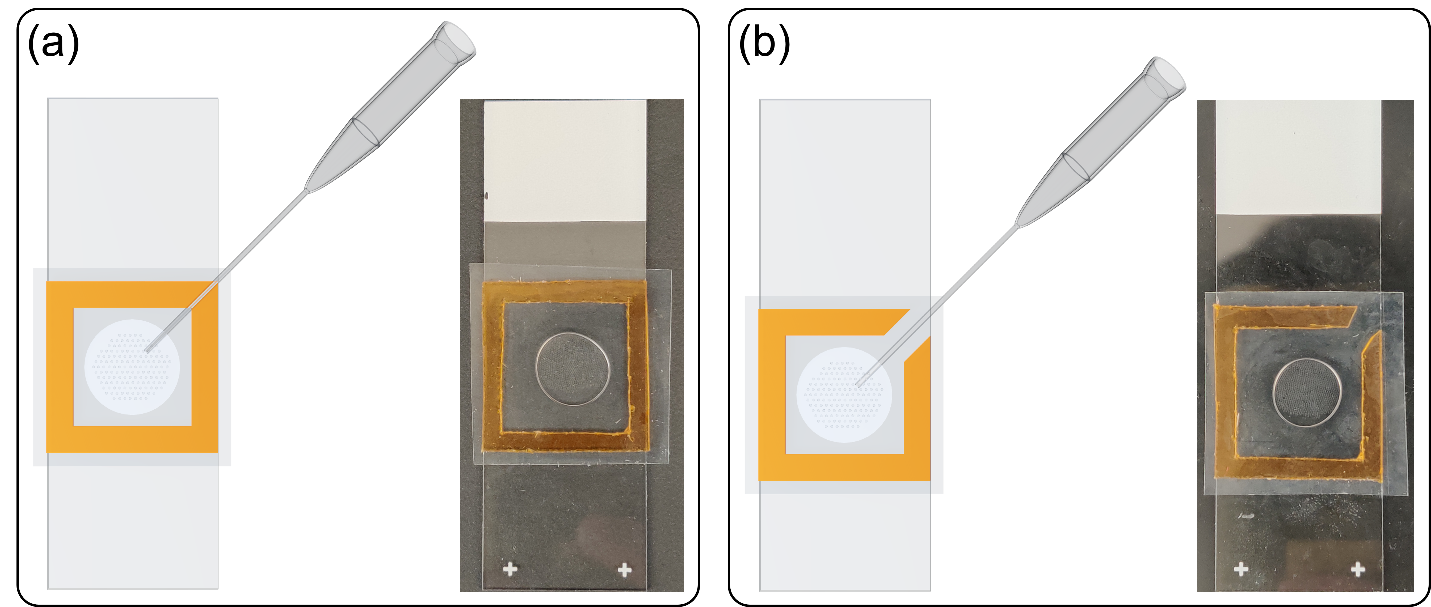


Figure S6. Droplet assembly and imaging chips made of double-sided tape on glass slides: (a) intact chip with droplets loaded from a corner by lifting the covering film at the corner up; (b) chip with a notch on the tape frame at a corner for inserting pipetting tips to load droplets. As long as the covering film is pressed down in the center to form a stable shallow center of the chip chamber, loaded droplets will be pinned in the center because of capillary pressure at the peripheral meniscus.


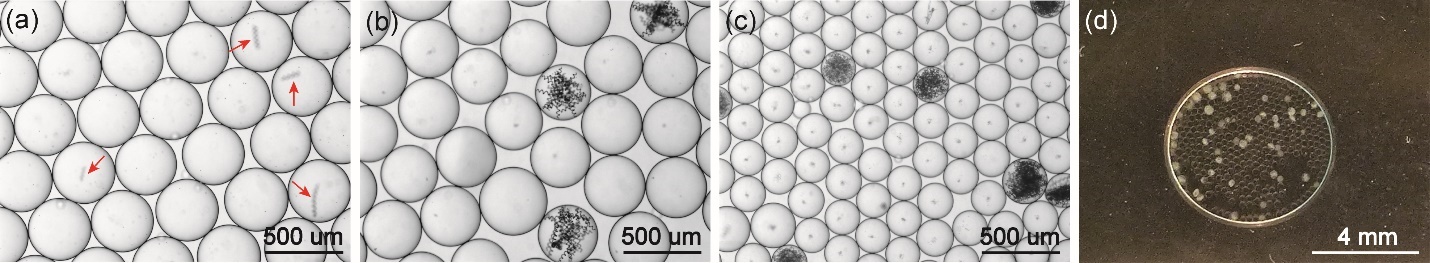


Figure S7. Photosynthetic culturing of spirulina in droplets loaded in a 200 µL PCR tube: (a) single spirulina in as-generated droplets; (b) proliferated spirulina in positive droplets after ten days of culturing; (c) spirulina almost occupies the whole positive droplets after 20 days of culturing; (d) smartphone imaging of the 20-day culturing droplets showing recognizable color/turbidity differences of positive droplets with spirulina.


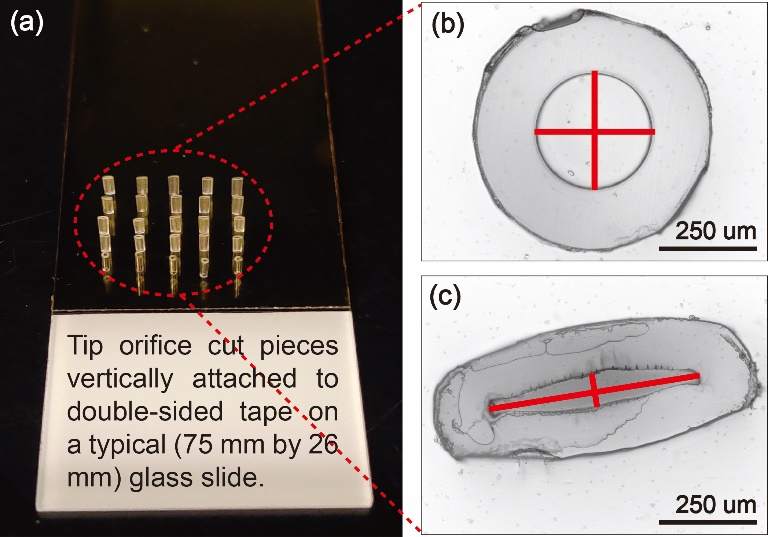


Figure S8. The estimation of tip orifice cross-section aspect ratio: (a) small pieces are cut from the head of the tips and then attached vertically to the double-sided tape covered on a regular glass slide; (b, c) example images of the cross-sections of tip cut pieces and the images are used to estimate the lengths of the long and short axes (red solid line segments, which are perpendicular to each other at the center point of the long line segment) to calculate the aspect ratios (the length of the long axis over the length of the short axis).


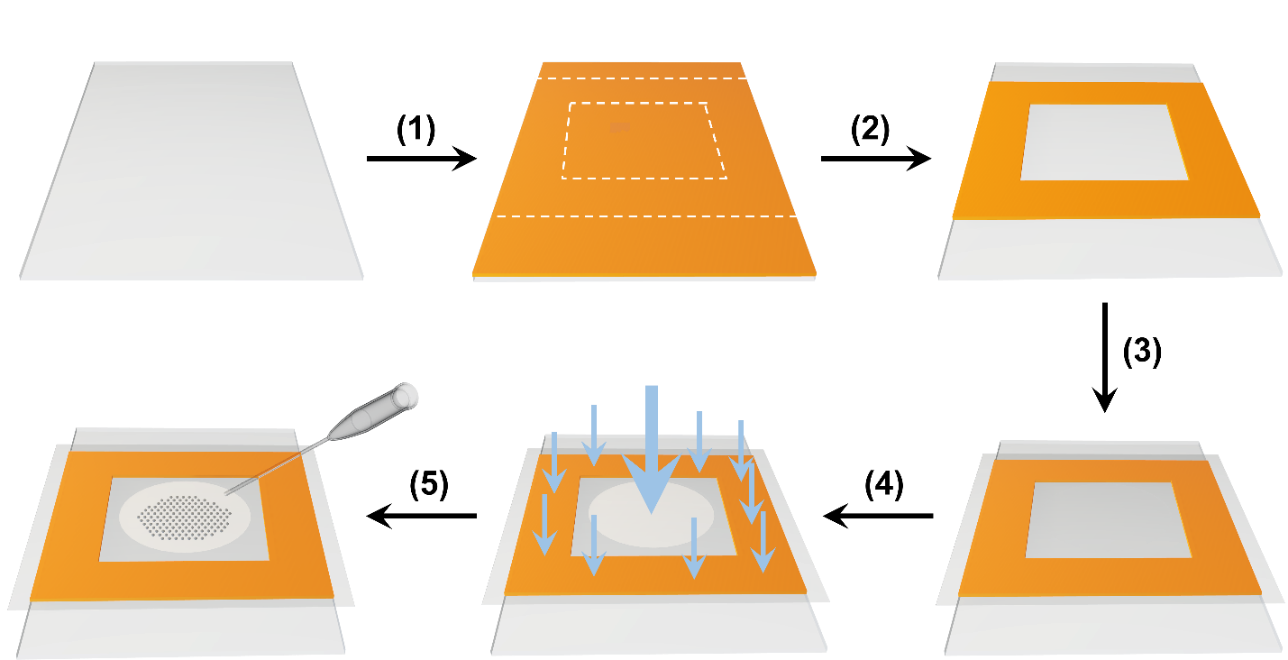


Figure S9. Procedures for making a tape-based droplet assembly and imaging chip: (1) Six pieces of double-sided polyimide tape are adhered to a glass slide layer by layer and the tape protection film (tape liner) of the last layer is left on the tape. (2) Chip dimension lines (white dash line) are drawn on the top protecting film, and a chip frame is cut out of the layered tape. (3) A new piece of tape protecting film slightly larger than the tape frame on the glass slide is used to cover the tape frame after alignment. (4) The center of the protection film is pressed onto the glass slide surface at the center of the tape frame and then the sides of the films are pressed tightly on the adhesive tape frame. (5) Droplets/oil are loaded via regular round orifice pipette from a corner by lifting the covering film a little.

**Brief video titles and legends for the supplementary videos:**

Video S1: 4X speed pipette droplet generation of 20 uL Water in 50 uL fluorinated oil: the video shows robust uniform droplet generation simply by manual pipetting with the deform pipette tip.

Video S2: 4X speed droplet loading to the imaging chip: the video shows how the droplets/oil are loaded into the tape-based imaging chip and how the loaded droplets/oil are immobilized in the shallow center of the chip.

Video S3. 4X speed droplet unloading from the imaging chip: the video shows how the droplets/oil are unload/removed/recycled from the tape-based imaging chip.
